# Porphyrin-copper (II) covalent organic framework nanoplatform with intrinsic bifunctional photodynamic and chemodynamic activities for synergistic multimodal cancer therapy

**DOI:** 10.1016/j.mtbio.2026.103579

**Published:** 2026-08-17

**Authors:** Yongjie Mo, Jie Hou, Hongli Li, Lijuan Wang, Linlu Zhao, Linhua Zhu, Chunyan Dai

**Affiliations:** a Key Laboratory of Tropical Medicinal Resource Chemistry of Ministry of Education, Key Laboratory of Tropical Medicinal Plant Chemistry of Hainan Province, The International Joint Research Center for Clean and Efficient Utilization of Hydrocarbon Resources in the South China Sea of Hainan Province, Engineering Research Center of Tropical Marine Functional Polymer Materials of Hainan Province, Key Laboratory of Water Pollution Treatment and Resource Reuse of Hainan Province, Key Laboratory of Functional Organic Polymers of Haikou, Hainan Normal University, Haikou, 571158, China; b Key Laboratory of Emergency and Trauma of the Ministry of Education, The First Affiliated Hospital, Hainan Medical University, Haikou, 571199, China; c Yazhou Bay Innovation Institute/College of Marine Science and Technology, Hainan Tropical Ocean University, Sanya, Hainan, 572022, China

**Keywords:** Covalent organic framework, Porphyrin, Photodynamic therapy, Chemodynamic therapy, Multimodal cancer therapy

## Abstract

Photodynamic therapy (PDT) efficacy is severely compromised by tumor hypoxia and the scarcity of efficient type-I photosensitizers, necessitating multimodal therapeutic strategies. Herein, we report a porphyrin-Cu (II) covalent organic framework (TBCOF) with intrinsic bifunctional photodynamic and chemodynamic activities. Sequential DOX encapsulation and hyaluronic acid (HA) surface functionalization afford HA-TBCOF@DOX, a tumor-targeted nanoplatform integrating four synergistic therapeutic modalities. Under 660 nm laser irradiation, the nanoplatform generates ROS signatures consistent with both type-I and type-II pathways for PDT, while the intrinsic Cu^2+^ centers are proposed to catalyze a Fenton-like reaction consistent with •OH generation for CDT. Concurrently, observations of O_2_ evolution suggest catalase-like activity that may decompose endogenous H_2_O_2_ into O_2_, alleviating hypoxia and potentiating oxygen-dependent PDT. Efficient photothermal conversion and pH-triggered DOX release contribute to photothermal and chemotherapeutic effects, respectively, while HA-mediated CD44 targeting enhances cellular uptake and therapeutic specificity. In vitro and in vivo studies demonstrate that laser-activated HA-TBCOF@DOX effectively suppresses tumor growth, induces apoptosis, and exhibits no apparent systemic toxicity under the tested experimental conditions. This work establishes a multifunctional COF-based nanoplatform that addresses PDT limitations observed in this study by integrating intrinsic PDT and CDT, providing a robust strategy for synergistic multimodal cancer therapy.

## Highlights

• Porphyrin-Cu(II) COF nanoplatform integrates PDT, CDT, PTT and chemotherapy.
• Synergistic type-I/II ROS generation with Cu^2+^ Fenton-like catalysis for oxidative stress.
• Catalase-mimetic H_2_O_2_ decomposition relieves hypoxia; pH-triggered DOX release.
• HA-CD44 targeting enhances internalization, achieving robust tumor suppression in vivo.

## Graphical abstract

**Figure:**
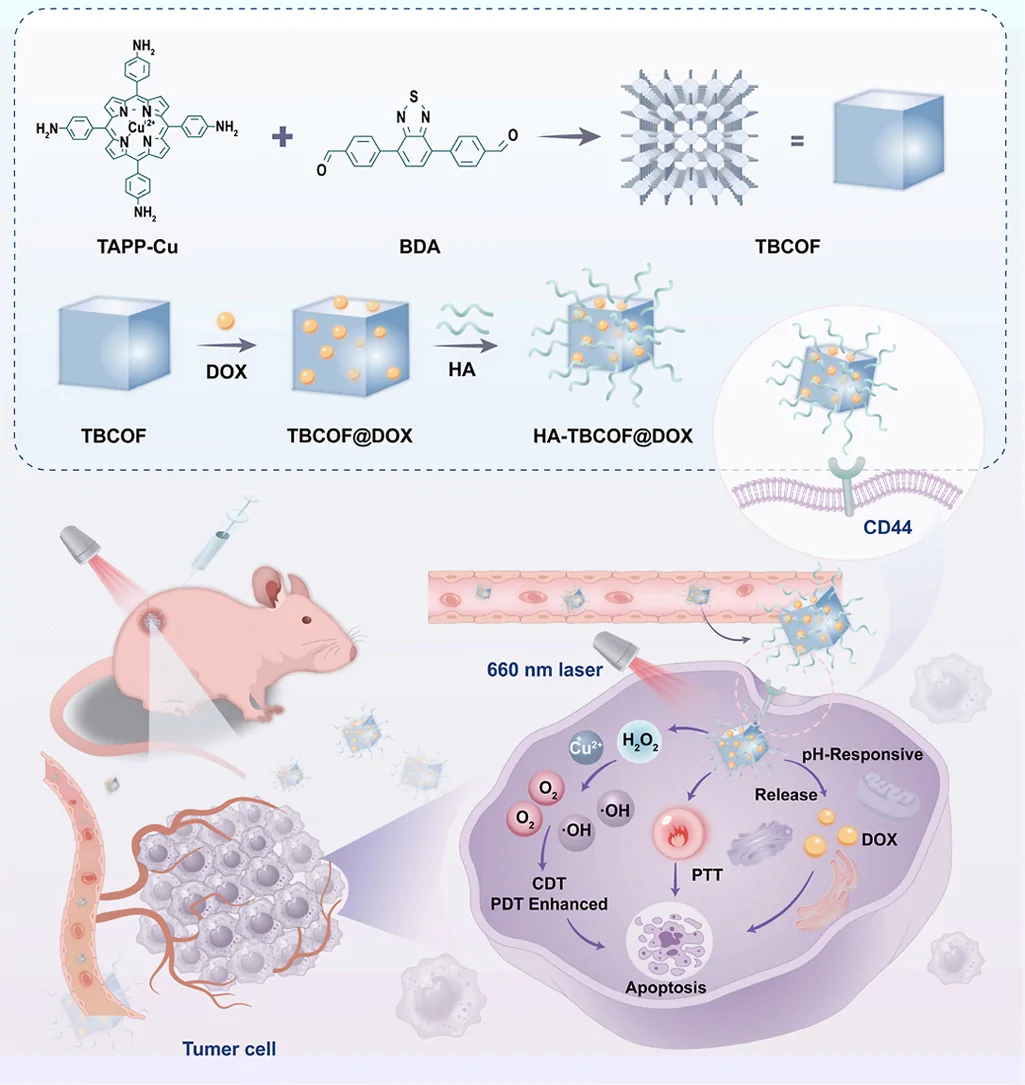

## 1 Introduction

Cancer remains a major cause of morbidity and mortality worldwide, with continuously rising incidence rates [1,2]. PDT has attracted considerable interest as a minimally invasive anticancer approach owing to its high spatiotemporal selectivity, low systemic toxicity, and compatibility with other treatment modalities [3,4]. Mechanistically, PDT relies on the photoexcitation of a photosensitizer (PS) to generate cytotoxic ROS that selectively damage tumor cells. Upon photoexcitation, the PS is promoted to its singlet excited state (S1) and subsequently undergoes intersystem crossing (ISC) to the triplet excited state (T_1_). From this state, two pathways can be followed: a type I route involving electron or proton transfer to produce superoxide (O_2_^−^•) and hydroxyl radicals (•OH), or a type II route that transfers energy to molecular oxygen, generating singlet oxygen (^1^O_2_) [[5], [6], [7]]. Most approved PDT protocols rely predominantly on type II photosensitizers. Their efficacy is, however, severely compromised in the hypoxic microenvironment of solid tumors, where limited O_2_ restricts ^1^O_2_ generation [8,9]. Type-I PDT can partially circumvent oxygen dependence through radical generation via electron transfer; however, existing type-I photosensitizers are limited by narrow absorption spectra and relatively low quantum yields [10,11]. Furthermore, complex tumor vasculature, robust antioxidant defense systems, and hypoxia-driven resistance collectively render single-modality PDT insufficient for complete tumor eradication [12]. The tumor microenvironment (TME), defined by hypoxia, immunosuppression, and metabolic reprogramming, fuels therapeutic resistance and tumor progression through malignant–stromal crosstalk [13,14]. TME-modulating strategies, including engineered extracellular vesicles and metal-phenolic network-mediated ferroptosis–immunotherapy cascades, can surmount these barriers by counteracting immunosuppression and potentiating antitumor immunity [15,16]. These limitations highlight the need for multimodal therapeutic strategies that combine PDT with complementary approaches.

Covalent organic frameworks (COFs) have emerged as a versatile class of crystalline porous polymers offering tunable pore structures, customizable chemical functionalities, and high surface areas [[17], [18], [19], [20]]. Incorporating porphyrin units as COF building blocks suppresses aggregation-caused quenching (ACQ) and preserves the photosensitizing activity of the porphyrin chromophores, making porphyrin-COFs attractive for drug delivery and phototherapy [[21], [22], [23], [24]]. For example, Zhang et al. constructed a DOX-loaded, HA-modified porphyrin COF for combined chemo-PDT [25], and Feng et al. reported an Fe-porphyrin COF nanozyme integrating catalytic therapy, PTT, and PDT [26]. Compared with liposomes, polymeric micelles, and amorphous mesoporous silica, porphyrin COFs deliver higher DOX loading via ordered pores and conjugated π–π interactions, and their rigid skeleton effectively suppresses premature drug leakage under physiological neutral conditions [22,27]. Covalently integrated porphyrin linkers eliminate aggregation-caused quenching and simultaneously generate dual type-I/II ROS, overcoming the photodynamic deactivation of photosensitizers physically loaded in conventional carriers [23]. However, most reported porphyrin COFs only perform oxygen-dependent type-II PDT without intrinsic catalytic capacity to alleviate tumor hypoxia [26]. Moreover, prior platforms introduce metal catalytic sites via post-synthetic doping instead of native porphyrin Cu^2+^ chelation, damaging crystallinity and triggering metal leakage, while most lack HA ligands to realize active tumor targeting [25,28]. Importantly, the electron-deficient benzothiadiazole moiety in the BDA linker serves as an ideal acceptor to construct a donor–acceptor system with the porphyrin donor, which narrows the bandgap, broadens visible-light harvesting, and facilitates charge separation for enhanced type-I/II ROS generation [[29], [30], [31]]. Nevertheless, the therapeutic performance of these systems remains constrained by tumor hypoxia and their predominant reliance on type II PDT. Importantly, existing porphyrin-based COF nanoplatforms have yet to exploit the intrinsic Cu^2+^-chelating capability of porphyrin units to enable CDT. Such an approach could simultaneously generate highly cytotoxic hydroxyl radicals (•OH) and appear to catalytically decompose endogenous H_2_O_2_ into O_2_, thereby alleviating tumor hypoxia and boosting PDT efficacy. Consequently, a porphyrin COF that intrinsically integrates both PDT and CDT functionalities remains largely unexplored yet highly desirable. In this context, COF-based nanoplatforms represent a promising avenue for developing synergistic multimodal anticancer strategies that integrate PDT with complementary therapeutic modalities [28,32,33]. However, the biomedical application of COFs is still at an early stage, and achieving both robust colloidal stability under physiological conditions and satisfactory long-term biosafety remains a critical bottleneck for their clinical translation [27].

**Scheme 1:**
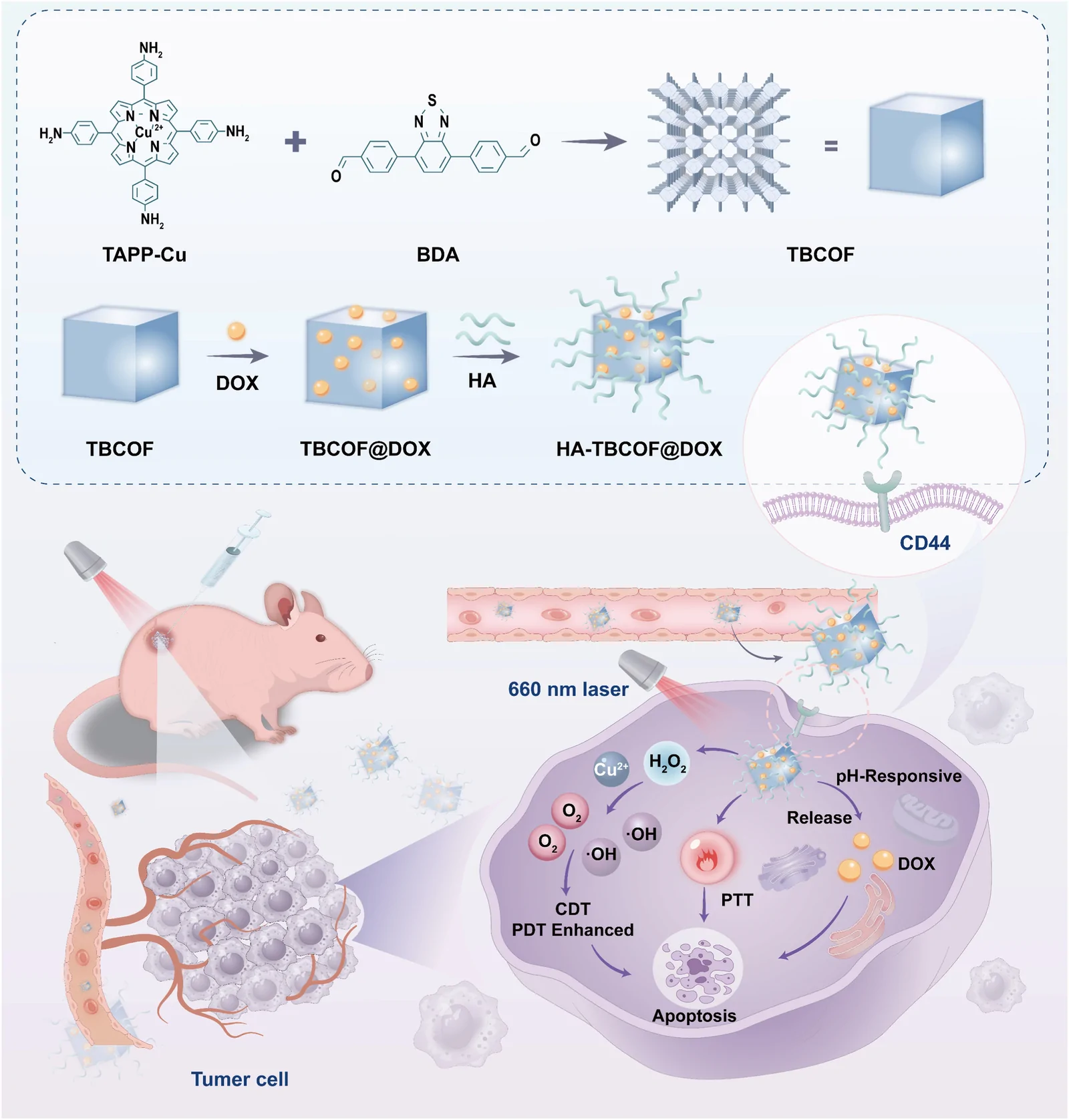
Schematic illustration of the HA-TBCOF@DOX nanoplatform for synergistic photodynamic, chemodynamic, photothermal, and chemotherapeutic multimodal cancer therapy.

Herein, we report a rationally designed porphyrin-based covalent organic framework featuring intrinsic photodynamic and chemodynamic activities. To further enhance tumor targeting and therapeutic efficacy, DOX was efficiently encapsulated into TBCOF, followed by covalent conjugation with HA, yielding the multifunctional nanoplatform HA-TBCOF@DOX. This system exhibits several distinct advantages, including exceptionally high drug loading capacity and pH-responsive DOX release, enabling on-demand chemotherapy within the acidic tumor microenvironment. Under 660 nm laser irradiation, HA-TBCOF@DOX simultaneously generates both type-I and type-II ROS, ensuring effective PDT. Meanwhile, the intrinsic Cu^2+^ centers are suggested to catalyze a Fenton-like reaction to convert endogenous H_2_O_2_ into highly toxic •OH for CDT, while concurrently producing O_2_ to relieve hypoxia and further potentiate PDT [23,34]. In addition, the nanoplatform displays efficient photothermal conversion, which acts synergistically with PDT and CDT to achieve enhanced therapeutic efficacy. The HA modification confers active targeting toward CD44-overexpressing cancer cells, significantly promoting cellular internalization and therapeutic specificity. In vitro studies demonstrate that HA-TBCOF@DOX, upon laser irradiation, markedly elevates intracellular ROS levels, induces mitochondrial membrane potential collapse, and triggers late-stage apoptosis, resulting in superior cytotoxicity compared to non-targeted counterparts. In vivo evaluation in a breast cancer xenograft model further reveals that laser-activated HA-TBCOF@DOX effectively suppresses tumor growth, reduces tumor burden, and induces extensive intratumoral apoptosis, while exhibiting negligible systemic toxicity, as evidenced by stable body weight. As illustrated in Scheme 1, this work establishes a multifunctional COF-based nanoplatform that seamlessly integrates targeted drug delivery, PDT, CDT, photothermal therapy, and chemotherapy. This integrated strategy provides a compelling route to mitigate the inherent limitations of monomodal PDT and achieve highly efficient and safe multimodal cancer therapy.

## 2 Materials and methods

### 2.1 Reagents and materials

4,4'-(benzo [c][1,2,5] thiadiazole-4,7-diyl) dibenzaldehyde (BDA) and Cu (II) 5,10,15,20-tetrakis (4-aminophenyl) porphyrin (TAPP-Cu, >95%) were purchased from Macklin Biochemical Co., Ltd. (Shanghai, China). Doxorubicin hydrochloride (DOX・HCl, >99%), n-butanol, and o-dichlorobenzene (o-DCB) were obtained from Sinopharm Chemical Reagent Co., Ltd. (Beijing, China). Glacial acetic acid (99.5%) was supplied by Tianjin Kemiou Chemical Reagent Co., Ltd. Hyaluronic acid (Moligand™ grade, extracted from cockscomb, MW = 800–1000 kDa) was obtained from Aladdin Biochemical Co., Ltd. (Shanghai, China).

For cell culture experiments, fetal bovine serum (FBS) was acquired from Every Green, while Dulbecco's Modified Eagle Medium (DMEM), phosphate-buffered saline (PBS), and trypsin were supplied by Solarbio. Fluorescent probes and assay kits (SOSG, DCFH-DA, MTT, Calcein-AM/PI, JC-1, Annexin V-FITC/PI) were purchased from Beyotime Biotechnology (Shanghai, China). All additional chemicals and reagents employed in this study were of analytical grade and utilized directly without any further purification.

### 2.2 Cell lines and animals

The 4T1 murine mammary carcinoma cell line was obtained from the Cell Bank of the Chinese Academy of Sciences (Shanghai, China) and cultured in high-glucose DMEM supplemented with 10% FBS and 1% penicillin-streptomycin at 37 °C in a humidified 5% CO_2_ atmosphere.

Female BALB/c nude mice (6–8 weeks, 18–20 g) were sourced from the Guangdong Medical Laboratory Animal Center (Guangdong, China). All animal procedures were approved by the Institutional Ethical Committee of Animal Experimentation at Hainan Medical University and performed in compliance with the NIH Guide for the Care and Use of Laboratory Animals.

### 2.3 Preparation of TBCOF

TBCOF was synthesized via solvothermal Schiff base condensation between BDA (13.77 mg, 0.04 mmol) and TAPP-Cu (14.73 mg, 0.02 mmol) in 1.1 mL of o-dichlorobenzene/n-butanol/6 M acetic acid (5:5:1, v/v/v). After 10 min of ultrasonication (40 kHz, 100 W) to dissolve monomers, the mixture was transferred to a 20 mL Pyrex tube, degassed by three freeze-pump-thaw cycles (liquid nitrogen freezing, vacuum < 10^−3^ Torr, room-temperature thawing), and flame-sealed under vacuum. The reaction was conducted at 120 °C for 72 h in an electric thermostatic drying oven. After the reaction, the resulting solid was collected and purified by repeated washing with tetrahydrofuran (THF) and dichloromethane (DCM). The crude product was isolated via centrifugation, resuspended in DCM, and further purified by vacuum filtration. The product was dried under vacuum at 60 °C for 12 h, yielding TBCOF as a purple-red flocculent solid (∼83% yield).

### 2.4 Preparation of TBCOF@DOX and HA-TBCOF@DOX

For DOX loading, 1 mg of TBCOF was dispersed in 1 mL of deionized water by 5 min bath sonication (40 kHz), then mixed dropwise with 1 mL DOX·HCl solution (2 mg mL^−1^, pH adjusted to 7.4 with 0.1 M NaOH) under magnetic stirring (600 rpm). The mixture was stirred in the dark at room temperature for 12 h to reach loading equilibrium. TBCOF@DOX nanoparticles were harvested by centrifugation (8000 rpm, 15 min), washed 3× with deionized water to remove unbound DOX, and resuspended in 2 mL deionized water. DOX loading capacity and encapsulation efficiency were calculated from supernatant absorbance at 480 nm, quantified against a pre-established calibration curve.

For HA functionalization, 500 μL of freshly prepared HA solution (15 mg mL^−1^) was added dropwise to the TBCOF@DOX dispersion under stirring (400 rpm) and reacted for 12 h in the dark. HA-TBCOF@DOX was collected by centrifugation (8000 rpm, 15 min), washed 2× with deionized water, and resuspended in pH 7.4 PBS to a stock concentration of 1 mg mL^−1^ (based on TBCOF content) for subsequent experiments.

### 2.5 Drug release profile of TBCOF@DOX

In vitro DOX release was evaluated by the dialysis method. Briefly, 5.0 mL TBCOF@DOX suspension (200 μg mL^−1^ DOX equivalent) was sealed in a dialysis bag (MWCO 3500 Da) and immersed in 40 mL PBS (pH 5.4 or 7.4). The system was incubated at 37 °C with orbital shaking at 600 rpm.

At preset time points (0, 1, 2, 4, 8, 12, 24, 36 h), 0.2 mL aliquots were withdrawn and replenished with an equal volume of pre-warmed fresh medium. Released DOX was quantified by absorbance at 480 nm using an Ultraviolet-visible Spectrophotometer. All experiments were performed in triplicate, and data are presented as mean ± SD.

### 2.6 Photodynamic properties of HA-TBCOF@DOX

The ROS generation capability of HA-TBCOF@DOX was evaluated by electron paramagnetic resonance (EPR) spectroscopy using specific spin-trapping agents. Typically, HA-TBCOF@DOX aqueous solution (100 μg mL^−1^) was mixed with 2,2,6,6-tetramethylpiperidine (TEMP) for singlet oxygen (^1^O_2_) detection, or with 5,5-dimethyl-1-pyrroline N-oxide (DMPO) for superoxide anion (O_2_^−^•) and hydroxyl radical (•OH) detection. The mixtures were irradiated with a 660 nm laser (1 W cm^−2^) for different time intervals (0, 1, and 5 min). EPR spectra were recorded immediately after irradiation at room temperature.

### 2.7 Photothermal properties of HA-TBCOF@DOX

To investigate the photothermal performance of HA-TBCOF@DOX, three sets of experiments were conducted using a thermometer for temperature recording. First, HA-TBCOF@DOX aqueous solutions at various concentrations (62.5, 125, 250, and 500 μg mL^−1^) were irradiated with a 660 nm laser at 1 W cm^−2^ for 15 min, and the temperature changes were monitored. Second, the TBCOF solution at 500 μg mL^−1^ was exposed to 660 nm laser irradiation at different power densities (0.5, 0.75, 1.0, and 1.25 W cm^−2^) for 15 min, with real-time temperature measurement. Third, the photothermal stability was evaluated by subjecting the TBCOF solution (500 μg mL^−1^) to four cycles of irradiation (660 nm, 1 W cm^−2^, 120 min), each followed by natural cooling without irradiation, during which the temperature profiles were continuously recorded. Photothermal conversion efficiency (η) was calculated by the Roper method from the cooling curve after laser shutoff, with details provided in the Supplementary Information.

### 2.8 Cellular uptake assay procedure

4T1 cells were plated into culture dishes at a density of 1 × 10^5^ cells per dish and cultured for 24 h to facilitate attachment. Cells were then incubated with free DOX, TBCOF@DOX, or HA-TBCOF@DOX (all 5 μg mL^−1^ DOX equivalent) for 4 h at 37 °C. For the HA-blocking group, cells were pre-incubated with 1 mg mL^−1^ free HA for 2 h before treatment. After treatment, nuclear staining was performed by adding Hoechst 33342 and incubating for an additional 10 min. Cellular uptake of DOX, TBCOF@DOX, and HA-TBCOF@DOX was subsequently examined using confocal laser scanning microscopy (CLSM).

### 2.9 Assessment of mitochondrial membrane potential

Mitochondrial membrane potential was assessed using a JC-1 probe. 4T1 cells were seeded in 6-well plates (1 × 10^5^ cells/well) and cultured for 24 h, then treated with control, TBCOF, TBCOF@DOX, HA-TBCOF@DOX, or HA-blocking + HA-TBCOF@DOX for 12 h. Where indicated, cells received 660 nm laser irradiation (1.0 W cm^−2^, 5 min) followed by a 2 h incubation. The cells were then stained with JC-1 working solution for 30 min, washed with PBS, and imaged using a fluorescence microscope.

### 2.10 Detection of intracellular ROS level

4T1 cells were seeded onto confocal dishes and incubated for 24 h to allow cell attachment. The cells were then treated with Control, TBCOF, TBCOF@DOX, or HA-TBCOF@DOX for 12 h. For hypoxia assays, cells were pre-equilibrated in 1% O_2_, 5% CO_2_, 94% N_2_ for 4 h and maintained under hypoxia throughout treatment and staining. Subsequently, the cells were exposed to 660 nm (1.0 W cm^−2^) laser irradiation for 5 min, followed by a 2 h incubation. The cells were then stained with 10 μM DCFH-DA for 20 min at 37 °C in the dark to detect intracellular reactive oxygen species (ROS). After gentle washing with PBS, the cells were counterstained with Hoechst 33342 (10 μg mL^−1^) for 10 min at 37 °C to visualize cell nuclei, followed by another gentle wash with PBS. Fluorescence images were then captured using an inverted fluorescence microscope, with green fluorescence indicating ROS levels and blue fluorescence indicating nuclear localization.

### 2.11 Cell viability detection

4T1 murine breast cancer cells were seeded into 96-well plates at a density of 5 × 10^3^ cells per well. For hypoxia assays, cells were pre-equilibrated in 1% O_2_, 5% CO_2_, 94% N_2_ for 4 h and maintained under hypoxia throughout treatment. Following a 24 h incubation to allow for cell attachment, the culture medium was replaced with fresh medium containing the test materials, and the cells were treated for 20 h. Where indicated, the cells were subsequently exposed to irradiation at 660 nm (1.0 W cm^−2^) for 5 min. After an additional 12 h recovery period, cell viability was assessed using the MTT assay. Specifically, 10 μL of MTT solution (5 mg mL^−1^) was added to each well, and the plates were incubated for 4 h to allow for the formation of formazan crystals. The resulting crystals were dissolved in 150 μL of dimethyl sulfoxide (DMSO), and the absorbance was measured at 570 nm using a microplate reader, with the signal intensity reflecting the relative viability of treated cells.

### 2.12 Live-cell imaging

For Calcein-AM and PI imaging, 4T1 cells were seeded into glass-bottom cell culture dishes at a density of 1 × 10^5^ cells per well. Following a 24 h incubation, the cells were treated with culture medium containing Control, TBCOF, TBCOF@DOX, or HA-TBCOF@DOX for 12 h. After laser irradiation (660 nm, 1.0 W cm^−2^, 5 min) followed by an additional 4 h incubation, the cells were stained with a working solution containing Calcein-AM and propidium iodide (PI) for 30 min. Fluorescence images were then captured using confocal laser scanning microscopy (CLSM).

### 2.13 Apoptosis analysis by flow cytometry

4T1 cells were plated in six-well plates and incubated for 24 h. Following treatment with Control, DOX, TBCOF@DOX, or HA-TBCOF@DOX for 12 h, the cells were subjected to laser irradiation (660 nm, 1.0 W cm^−2^, 5 min) and subsequently cultured for another 4 h. After three washes with PBS, the cells were harvested using EDTA-free trypsin and collected via centrifugation. Staining was performed using an Annexin V-FITC/PI apoptosis detection kit according to the manufacturer's protocol, and the stained cells were analyzed by flow cytometry.

### 2.14 Evaluation of antitumor efficacy in vivo

The 4T1 tumor-bearing mouse model was established by subcutaneously injecting 4T1 cells (1 × 10^6^) into the right dorsal flank of female BALB/c nude mice. When the tumor volume reached approximately 100 mm^3^, the tumor-bearing mice were randomly divided into four groups (n = 5): Control, TBCOF, TBCOF@DOX, or HA-TBCOF@DOX. Subsequently, the mice received five intratumoral injections of the corresponding formulations on days 0, 3, 6, 9, and 12. Immediately after each injection, laser irradiation (660 nm, 0.5 W cm^−2^, 5 min) was applied to the designated groups. Tumor volumes and body weights were recorded every other day. Tumor volume was calculated using the formula: 0.5 × L × W^2^, where V represents tumor volume, and L and W denote the length and width of the tumor, respectively. After 15 days of treatment, all mice were euthanized by cervical dislocation, and their tumors were collected for histological sectioning.

### 2.15 Statistical analysis

All data were presented as the mean ± SEM. Statistical analyses were carried out using GraphPad Prism 10. For comparisons between two groups (e.g., laser-irradiated vs. non-irradiated at a given concentration), an unpaired two-tailed Student's t-test was employed. For comparisons involving three or more groups, one-way or two-way analysis of variance (ANOVA) followed by Tukey's multiple comparisons test was used. Statistical significance is defined as *p < 0.05, **p < 0.01, and ***p < 0.001.

## 3 Results and discussion

### 3.1 Preparation and characterization

**Fig. 1:**
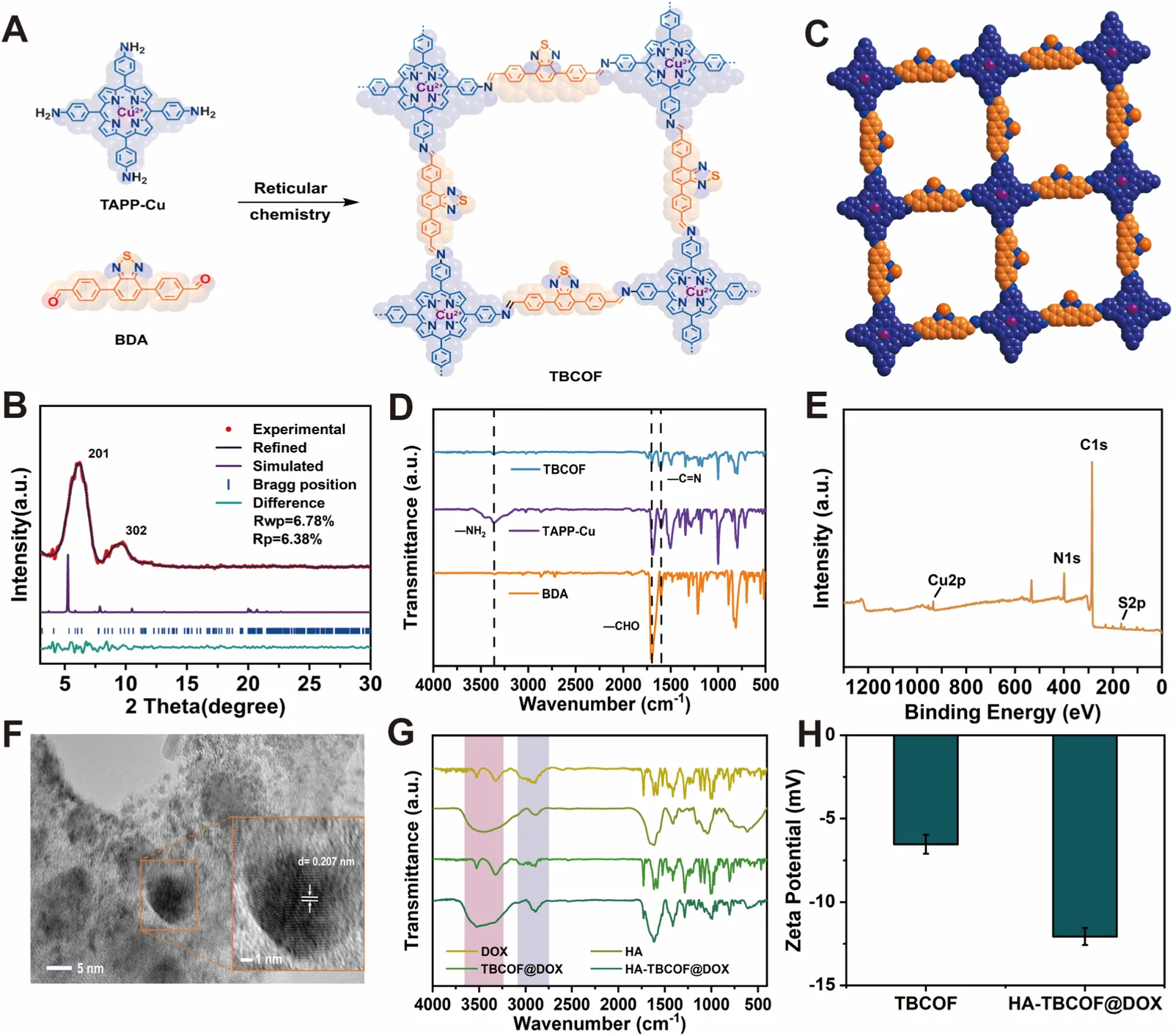
Synthesis, structural characterization, and surface properties of TBCOF and its drug-loaded derivatives. (A) Synthetic route and chemical structure of TBCOF. (B) Eclipsed AA-stacking mode of TBCOF. (C) Experimental PXRD pattern with Pawley refinement (Rwp = 6.78%, Rp = 6.38%). (D) FT-IR spectra of TAPP-Cu, BDA, and TBCOF. (E) XPS survey spectrum of TBCOF confirming C, N, S, and Cu. (F) HR-TEM image showing lattice fringes with an interplanar distance of approximately 0.207 nm. (G) FT-IR spectra of TBCOF@DOX and HA-TBCOF@DOX. (H) Zeta potentials of TBCOF and HA-TBCOF@DOX (mean ± SD, n = 3).

Under the classical solvothermal synthesis, the target TBCOF was synthesized via a vacuum-assisted solvothermal polycondensation of 5,10,15,20-Tetrakis-(4-aminophenyl)-porphyrin-Cu-(II) (TAPP-Cu) and 4,4'-(benzo[c] [1,2,5]thiadiazole-4,7-diyl)dibenzaldehyde (BDA) in a ternary solvent system consisting of o-dichlorobenzene, n-butanol, and aqueous acetic acid. The reaction was carried out at 120 °C for three days, after which the resulting precipitate was collected and further dried under vacuum to yield a purple-red flocculent solid. The synthetic route and chemical structure of the TBCOF are illustrated in Fig. 1A. To verify the crystalline structure, powder X-ray diffraction (PXRD) was employed. The diffraction pattern exhibited a strong peak at 6.16° and a moderate peak at 9.51°, corresponding to distinct characteristic reflections (Fig. 1C). Structural simulation was conducted using Materials Studio software, and the simulated PXRD pattern showed excellent agreement with the experimental results. Pawley refinement yielded low residual factors (Rwp = 6.78%, Rp = 6.38%), with the observed peaks indexed to the (201) and (302) lattice planes, respectively. Notably, the TBCOF adopts a typical two-dimensional layered framework with an eclipsed AA-stacking mode, as evidenced by the consistency between the experimental peak positions and the theoretical pattern (Fig. 1B). The successful condensation was further confirmed by Fourier transform infrared (FT-IR) spectra. As depicted in Fig. 1D, the spectrum of TAPP-Cu displayed a stretching vibration at 3362 cm^−1^ corresponding to N–H bonds, while BDA exhibited a strong band at 1705 cm^−1^ attributable to C=O bonds. In the FT-IR spectrum of TBCOF, the emergence of a new absorption band at 1606 cm^−1^, characteristic of C=N bonds, along with the disappearance of the N–H and C=O stretching bands, confirmed the successful Schiff base condensation between the aldehyde and amine groups. X-ray photoelectron spectroscopy (XPS) was employed to elucidate further the elemental composition and chemical bonding states of TBCOF. The survey spectrum confirmed the coexistence of C, N, S, and Cu (Fig. 1E), consistent with the expected framework architecture. High-resolution XPS analysis provided detailed insights into the valence states and bonding configurations. The C 1s spectrum (Fig. S1) revealed two characteristic peaks at 284.80 eV and 287.13 eV, corresponding to C–C and C–O species, respectively. The N 1s spectrum (Fig. S2) was deconvoluted into two distinct components at 397.83 eV and 400.19 eV, attributable to imine (C=N) and amine (C–N) moieties. In the S 2p region (Fig. S3), two prominent peaks were observed at 164.33 eV and 165.62 eV, assigned to S 2p_3/2_ and S 2p_1/2_, respectively. The Cu 2p spectrum (Fig. S4) resolved four components: Cu 2p_3/2_ at 933.69 eV, a satellite peak at 942.00 eV corresponding to Cu^2+^, and Cu 2p_1/2_ signals at 953.54 eV and 961.85 eV. Collectively, these XPS results corroborated the successful formation of the target COF structure. The permanent porosity of the COF was evaluated by collecting N_2_ adsorption and desorption isotherms at 77 K on samples activated through degassing under high vacuum at 120 °C before measurement. Brunauer-Emmett-Teller (BET) analysis of the adsorption data yielded a specific surface area of 139.3 m^2^ g^−1^ (Fig. S5). Pore size distribution, derived from non-local density functional theory (NL-DFT), revealed a narrow distribution centered at a pore diameter of 4.220 nm, with a total pore volume of 0.143 cm^3^ g^−1^ (Fig. S6). Additionally, the HR-TEM micrographs (Fig. 1F and S7) revealed well-defined lattice fringes, corresponding to an interplanar distance of approximately 0.207 nm. Together, these results confirm the formation of a crystalline COF with permanent mesoporosity, as evidenced by its moderate surface area, narrow pore size distribution, and well-defined lattice fringes observed by HR-TEM.

Following the characterization of TBCOF, FT-IR analysis further substantiated the successful preparation of TBCOF@DOX and HA-TBCOF@DOX. The FT-IR spectrum of TBCOF@DOX exhibited characteristic peaks corresponding to both TBCOF and DOX, confirming efficient drug loading. Following hyaluronic acid modification, new peaks characteristic of HA appeared, demonstrating effective surface functionalization (Fig. 1G). Collectively, these spectroscopic findings confirm the successful construction of both nanoplatforms. Morphological characterization by SEM revealed that TBCOF consisted of uniform nanoparticles with a size of approximately 100 nm, which agglomerated into a powder-like morphology (Fig. S8). After DOX loading and HA modification, HA-TBCOF@DOX maintained similar morphologies with slightly increased particle sizes (Fig. S9). EDS elemental mapping confirmed the homogeneous distribution of C, N, O, and Cu within TBCOF. In HA-TBCOF@DOX, the C and N signals from DOX and HA appeared with enhanced intensity, further supporting successful drug loading and surface functionalization (Fig. S10 and S11). Furthermore, Dynamic light scattering (DLS) analysis indicated that TBCOF maintained a uniform dispersion under both physiological and acidic conditions, with mean hydrodynamic diameters of 283.9 nm at pH 7.4 and 274.5 nm at pH 5.4 (Fig. S12 and S13). The particle size remained nearly unchanged over six days across pH 7.4, pH 5.4, and 10% FBS/PBS media (Fig. S14), demonstrating excellent colloidal stability and robust resistance to serum protein-induced aggregation. Across varying ionic strengths, the diameter increased only marginally from ∼270 to ∼300 nm (Fig. S15), confirming that the covalent COF framework effectively prevents electrolyte-mediated destabilization. Following DOX loading and subsequent HA modification, the resulting HA-TBCOF@DOX complex exhibited a moderate increase in hydrodynamic diameter to 401.5 nm (Fig. S16), consistent with effective drug encapsulation. Moreover, zeta potential measurements revealed a significant shift from −6.53 mV for unmodified TBCOF to −12.07 mV for HA-TBCOF@DOX (Fig. 1H), supporting successful surface functionalization. Notably, HA modification reduced protein corona formation by ∼27% relative to bare TBCOF (Fig. S17), which is anticipated to attenuate mononuclear phagocyte system clearance and prolong circulation for enhanced tumor accumulation via both passive EPR and active CD44-mediated targeting. Collectively, these results demonstrate the robust stability, favorable surface properties for prolonged circulation, and translational potential of the TBCOF platform under physiologically relevant conditions. Thermogravimetric analysis (TGA) revealed that TBCOF and HA-TBCOF@DOX possessed good thermal stability, with onset decomposition temperatures of 340.17 °C and 329.01 °C, respectively (Fig. S18).

### 3.2 Phototherapeutic properties and drug release

**Fig. 2:**
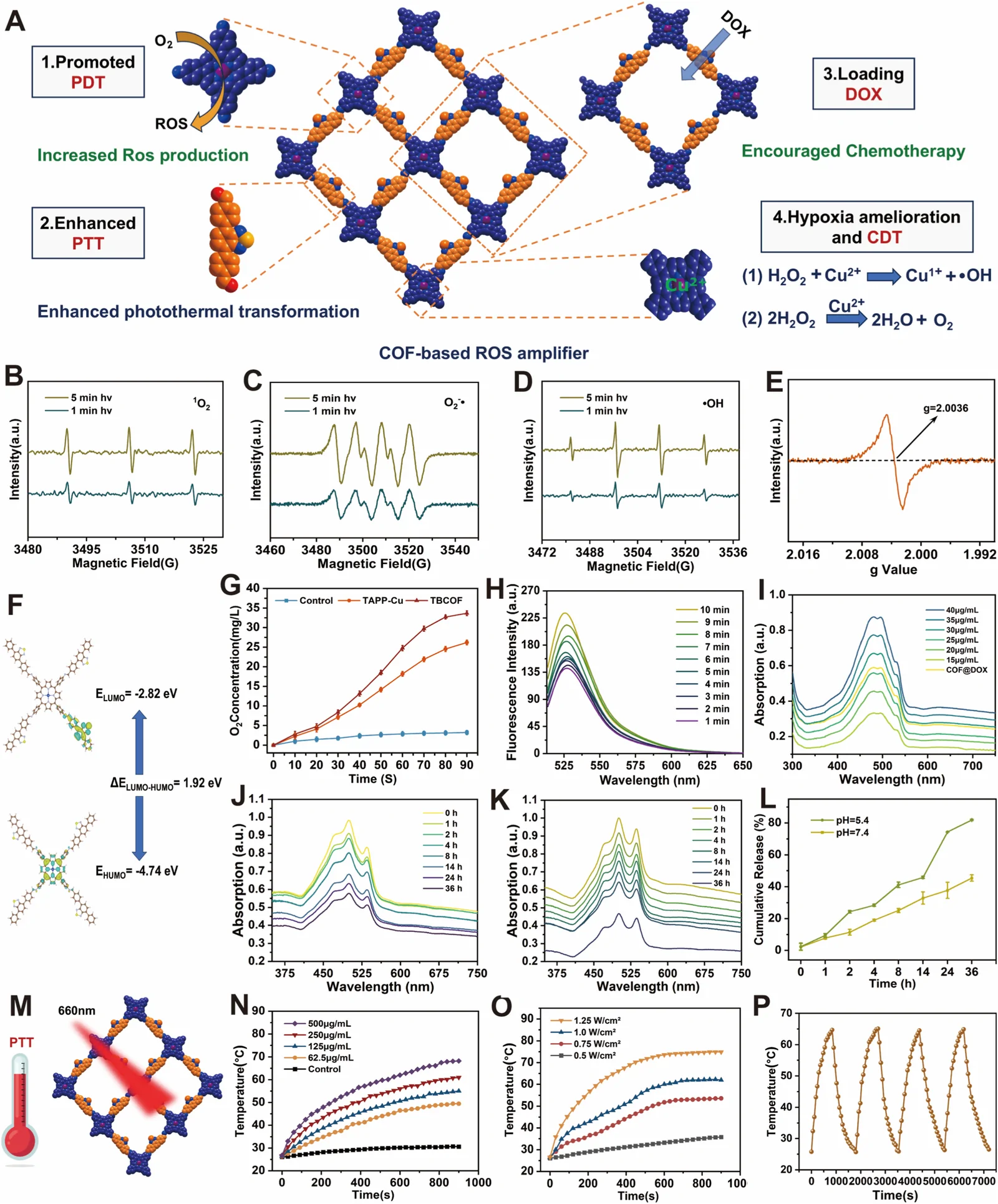
Phototherapeutic properties, ROS generation, drug release, and photothermal performance of HA-TBCOF@DOX and TBCOF. (A) Multifunctional features of TBCOF: ROS generation, drug loading, PTT, and catalytic H_2_O_2_ decomposition to O_2_. (B–D) EPR spectra of ^1^O_2_, O_2_^−^•, and •OH from TBCOF with increasing irradiation time. (E) EPR spectrum of oxygen vacancies in TBCOF. (F) DFT-calculated HOMO-LUMO band gap of TBCOF. (G) Catalytic H_2_O_2_ decomposition by TBCOF generating dissolved O_2_ over time (mean ± SD, n = 3). (H) SOSG fluorescence intensity of ^1^O_2_ from HA-TBCOF@DOX under a 660 nm laser (0–10 min). (I) UV-Vis spectrum for DOX loading efficiency. (J, K) DOX release from TBCOF@DOX at pH 7.4 and 5.4 over 48 h. (L) Cumulative DOX release at pH 5.4 and 7.4 over 48 h. (M) Schematic of the photothermal effect of TBCOF under a 660 nm laser (mean ± SD, n = 3). (N) Photothermal heating curves of HA-TBCOF@DOX at different concentrations under a 660 nm laser. (O) Temperature elevation of HA-TBCOF@DOX at a fixed concentration under different laser powers. (P) Cyclic heating-cooling curves of HA-TBCOF@DOX showing photothermal stability.

As a well-established photosensitizer, porphyrin has been clinically applied in PDT for years [[35], [36], [37]]. Leveraging the porphyrin-mediated optical properties and hierarchical porous architecture of TBCOF, we anticipated that this material would achieve enhanced ROS generation along with high drug-loading efficiency (Fig. 2A). Electron paramagnetic resonance (EPR) spectroscopy with specific spin traps was employed to evaluate ROS generation by TBCOF under 660 nm irradiation. As shown in Fig. 2B, a characteristic triplet signal (1:1:1) of TEMP-trapped ^1^O_2_was observed. Parallel DMPO trapping experiments revealed the production of O_2_^−^• (1:1:1:1 quartet) and •OH (1:2:2:1 quartet) (Fig. 2C and D). All three ROS signals intensified with prolonged irradiation, indicating time-dependent accumulation. The concurrent Type-I/II ROS generation is inferred from characteristic TEMP and DMPO spectral patterns under the present experimental conditions. The co-detection of Type I-associated (O_2_^−^• and •OH) and Type II-associated (^1^O_2_) species supports a dual-pathway mechanism, with the Type I contribution proposed to arise from abundant oxygen vacancies (g = 2.0036, Fig. 2E). DFT calculations revealed a narrow HOMO–LUMO gap of 1.92 eV for TBCOF (Fig. 2F), which enables efficient visible-light absorption and favorable photogenerated carrier separation [[38], [39], [40]]. This low bandgap provides the thermodynamic driving force for sustained generation of photoelectrons and holes, directly accounting for the progressive increase in ROS signals over time. The EPR-detected rise in ^1^O_2_, O_2_^−^•, and •OH further confirms efficient energy/electron transfer from the COF framework to molecular oxygen. Thus, combining EPR and DFT results, TBCOF serves as an efficient multi-ROS generator, where the porphyrin unit and oxygen vacancies work synergistically. Notably, even in the absence of light, EPR analysis revealed a distinct •OH signal upon addition of H_2_O_2_, consistent with TBCOF mediating CDT via a Cu^2+^-associated Fenton-like process (Fig. S19). Additionally, under conditions mimicking the tumor microenvironment, real-time monitoring of dissolved oxygen using a dissolved oxygen probe revealed that TBCOF promotes decomposition of endogenous H_2_O_2_ into O_2_, suggesting catalase-like activity (Fig. 2G). This capacity to alleviate tumor hypoxia holds significant promise for enhancing oxygen-dependent therapies such as photodynamic therapy. The generation of ^1^O_2_ was quantitatively evaluated using the SOSG fluorescent probe. Under 660 nm laser irradiation (0–10 min), both the porphyrin-based monomer TAPP-Cu and TBCOF exhibited time-dependent increases in fluorescence intensity, with longer illumination leading to stronger signals (Fig. S20 and S21). Notably, TBCOF demonstrated markedly superior ^1^O_2_ production capability compared to its monomeric counterpart. Furthermore, under tumor-mimicking conditions containing H_2_O_2_, the photosensitizing performance of TBCOF was further enhanced, showing a significant increase in fluorescence intensity relative to pristine TBCOF under the same irradiation conditions (Fig. 2H). These results highlight the excellent ROS-generating efficiency of TBCOF, particularly in a tumor-relevant oxidative environment.

The specific surface area and aromatic framework of TBCOF offer abundant binding sites and strong π-π stacking for DOX loading. To optimize drug loading, 1 mg of TBCOF was mixed with 2 mg of DOX in 1 mL of water under ultrasonication for 12 h. The resulting suspension was transferred into a dialysis bag (MWCO 3500 Da) and dialyzed against 4 L of deionized water for 48 h, with the water renewed every 4 h, yielding TBCOF@DOX. UV-Vis spectrophotometry at 480 nm (Fig. 2I) gave a drug encapsulation efficiency of 66.24%, derived from a calibration curve with R^2^ > 0.9992 (Fig. S22). Release behavior was then examined under conditions mimicking normal physiology (pH 7.4) and the tumor microenvironment (pH 5.4). The DOX-specific absorbance was recorded over 48 h (Fig. 2J and K). After 48 h, cumulative release reached 81.89% at pH 5.4 and 45.51% at pH 7.4 (Fig. 2L), with moderately accelerated release at acidic pH, likely attributable to protonation-induced weakening of π-π stacking between DOX and the COF [[41], [42], [43]]. FT-IR spectra of TBCOF@DOX before and after release at pH 5.4 (Fig. S23) showed no evident shift or new bands, confirming the framework's chemical stability and structural integrity under acidic conditions.

The photothermal performance of HA-TBCOF@DOX was evaluated under 660 nm laser irradiation (Fig. 2M). Aqueous dispersions (62.5–500 μg mL^−1^) exhibited a rapid temperature elevation when exposed to 1 W cm^−2^ for 900 s. At a concentration of 500 μg mL^−1^, the peak temperature reached 68.2 °C, representing a 37.6 °C increase above the water control (Fig. 2N). The photothermal response was further examined across laser intensities from 0.5 to 1.25 W cm^−2^, attaining 74.9 °C at the highest power density (Fig. 2O). Digital photographs captured a time-dependent temperature elevation in the HA-TBCOF@DOX group compared to the water control under 1.0 W cm^−2^ irradiation over 10 min, with quantified thermal intensities confirming marked photothermal conversion (Fig. S24 and S25). Four consecutive heating–cooling cycles showed almost no decrease in heating capacity, confirming the material's photostability and reusability (Fig. 2P). The photothermal conversion efficiency (η) was calculated as 54.0% from the linear fitting of the cooling period (Fig. S26 and S27). Collectively, these results underscore the efficient and stable photothermal conversion capability of HA-TBCOF@DOX, supporting its potential for laser-induced tumor therapy.

### 3.3 In vitro antitumor activity of HA-TBCOF@DOX

**Fig. 3:**
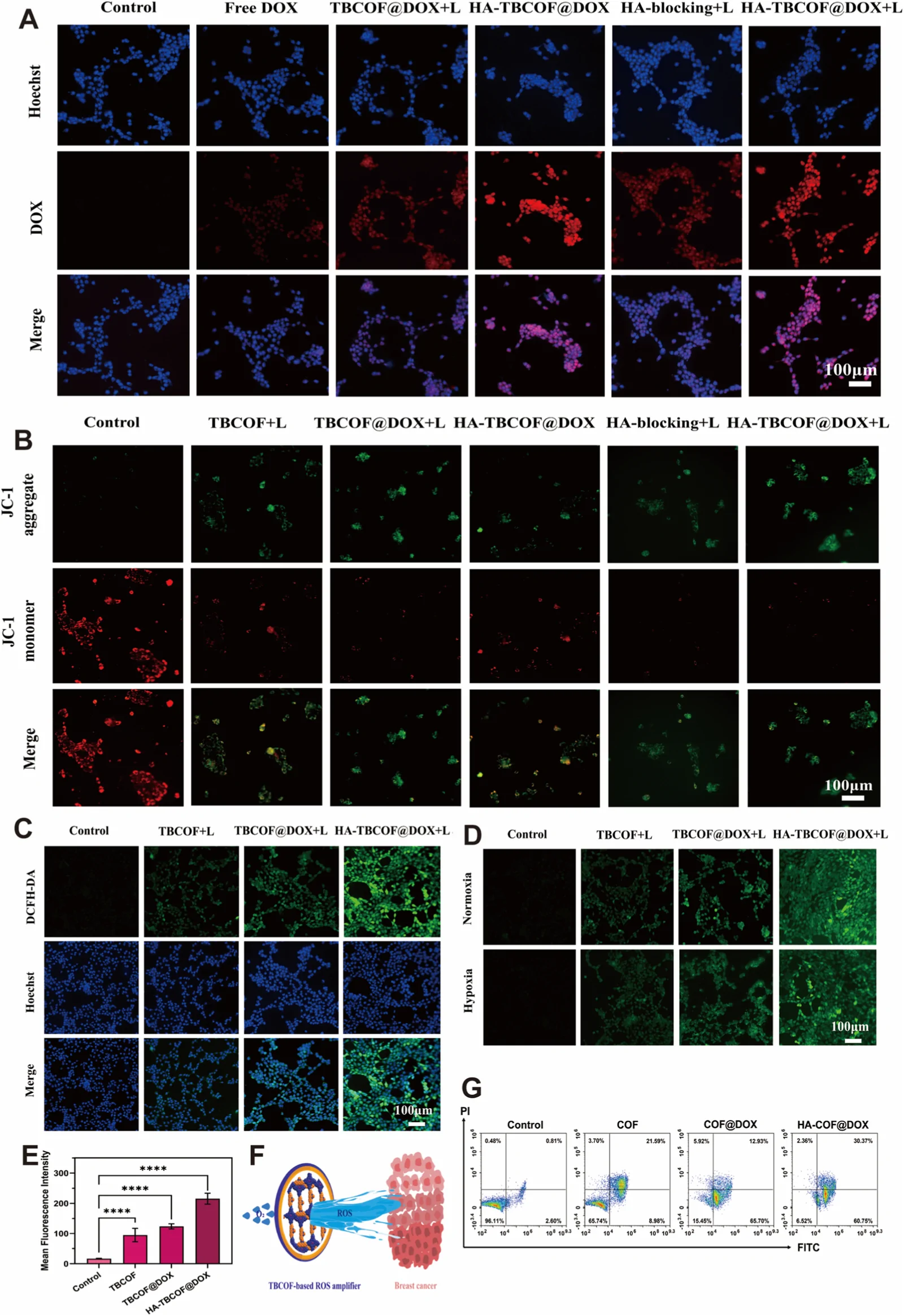
In vitro cellular uptake, mitochondrial membrane potential, ROS generation, and apoptosis of HA-TBCOF@DOX in 4T1 cells. (A) CLSM images of cellular uptake after 4 h incubation with DOX, TBCOF@DOX, HA-TBCOF@DOX, or HA-blocking + HA-TBCOF@DOX (scale bar: 100 μm). (B) JC-1 staining for mitochondrial membrane potential (ΔΨm) in 4T1 cells after treatment with TBCOF + L, TBCOF@DOX + L, HA-TBCOF@DOX, HA-blocking + L, or HA-TBCOF@DOX + L (scale bar: 100 μm). (C) DCFH-DA staining for ROS generation in different treatment groups under laser irradiation (scale bar: 100 μm). (D) Representative CLSM images of DCFH-DA staining for intracellular ROS generation in 4T1 cells under normoxia or hypoxia (1% O_2_) after various treatments with laser irradiation (scale bar: 100 μm). (E) Quantitative analysis of ROS fluorescence intensity (Statistical significance was determined by one-way ANOVA followed by Tukey's multiple comparisons test, *p < 0.05, **p < 0.01, ***p < 0.001). (F) Schematic illustration of ROS-mediated suppression of breast tumor cells by HA-TBCOF@DOX. (G) Flow cytometry analysis of Annexin V-FITC/PI-stained cells after various treatments with laser irradiation.

The targeting ability of HA-TBCOF@DOX arises from specific recognition between hyaluronic acid and CD44 receptors, which are overexpressed on the surface of many cancer cells [[44], [45], [46]]. CLSM was used to examine cellular uptake of DOX, TBCOF@DOX, and HA-TBCOF@DOX in 4T1 cells. As shown in Fig. 3A, free DOX exhibited weak fluorescence, while TBCOF@DOX + L and Free HA pre-incubation + HA-TBCOF@DOX + Laser (HA-blocking + L) showed comparable moderate fluorescence, indicating limited uptake enhancement by passive encapsulation or competitive CD44 blockade. In contrast, HA-TBCOF@DOX and HA-TBCOF@DOX + L displayed markedly stronger and comparable fluorescence, consistent with HA-mediated targeting. The attenuated fluorescence in the HA-blocking + L group, approaching that of TBCOF@DOX + L, directly validates specific HA-CD44 recognition. These results indicate that DOX encapsulation in TBCOF enhances uptake over free drug, and HA coating amplifies this via active targeting, which is competitively abrogated by excess free HA. Building on this targeting efficiency, mitochondrial membrane potential (ΔΨm), a critical factor in early apoptosis, was investigated [47,48]. 4T1 cells were treated with TBCOF + L, TBCOF@DOX + L, HA-TBCOF@DOX, HA-blocking + L, or HA-TBCOF@DOX + L for 12 h, followed by 660 nm laser irradiation for 5 min where indicated, and stained with JC-1. Brightfield images confirmed comparable cell density across all groups under white-light illumination (Fig. S28), excluding the possibility that fluorescence variations arise from differences in cell number rather than genuine ΔΨm changes. As shown in Fig. 3B, TBCOF + L and HA-TBCOF@DOX induced modest ΔΨm reduction, while TBCOF@DOX + L and HA-blocking + L exhibited moderate depolarization. Notably, HA-TBCOF@DOX + L showed the most pronounced ΔΨm disruption, with diminished red aggregates and enhanced green monomers, indicating significant apoptosis. The comparable ΔΨm loss in TBCOF@DOX + L and HA-blocking + L confirms that compromised uptake due to CD44 blockade directly diminishes apoptosis. Collectively, HA-mediated targeting enhances internalization and translates into superior mitochondrial dysfunction and apoptotic efficacy upon laser activation, critically dependent on specific HA-CD44 recognition.

Given the excellent targeting performance of HA-TBCOF@DOX, we further investigated its ROS generation capability under both normoxic and hypoxic conditions. The DCFH-DA probe was used to evaluate ROS production, followed by quantitative analysis. CLSM images revealed pronounced green fluorescence in the TBCOF@DOX + L and HA-TBCOF@DOX + L groups under light irradiation, with the latter exhibiting the most intense signal. In contrast, negligible fluorescence was observed in the control group, while the TBCOF + L group showed moderate fluorescence (Fig. 3C). Notably, under hypoxic conditions, both TBCOF + L and TBCOF@DOX + L maintained considerable ROS generation, with fluorescence intensities comparable to their normoxic counterparts (Fig. 3D). This suggests that the Type I ROS pathway (O_2_^−^• and •OH) and intrinsic CDT activity of TBCOF are less reliant on molecular oxygen and remain effective in the hypoxic tumor microenvironment. In contrast, HA-TBCOF@DOX + L exhibited the strongest fluorescence under both normoxia and hypoxia, with the hypoxic signal only marginally attenuated relative to normoxia, further underscoring the oxygen-independent nature of its therapeutic mechanism. Fluorescence intensity line profiles across individual cells confirmed that the HA-TBCOF@DOX + L group displayed markedly higher DCFH-DA signals colocalized with nuclear regions compared to TBCOF@DOX + L, directly reflecting enhanced intracellular ROS accumulation via HA-mediated targeting (Fig. S29–S32). Quantitative analysis demonstrated that HA-TBCOF@DOX + L produced significantly higher ROS levels than all other groups under normoxic conditions, which arises from the superior cellular delivery efficiency of HA-TBCOF@DOX in 4T1 cells (Fig. 3E). The superior cellular delivery efficiency of HA-TBCOF@DOX led to greater ROS generation in 4T1 cells (Fig. 3F). Collectively, these results indicate that HA-TBCOF@DOX markedly enhances ROS production upon light exposure, highlighting the great potential of targeted PDT for tumor suppression. To evaluate the apoptotic effects of different treatments on 4T1 cells, Annexin V-FITC/PI double staining followed by flow cytometry was performed. As shown in Fig. 3G, the control group contained most cells in the bottom left quadrant (live cells), indicating minimal apoptosis. In contrast, treatment with TBCOF, TBCOF@DOX, or HA-TBCOF@DOX combined with laser irradiation resulted in a substantial increase in cells in both the bottom right (early apoptosis) and top right (late apoptosis) quadrants. Notably, among all treated groups, HA-TBCOF@DOX under light exposure induced the highest proportion of late apoptotic cells, demonstrating its superior pro-apoptotic activity.

**Fig. 4:**
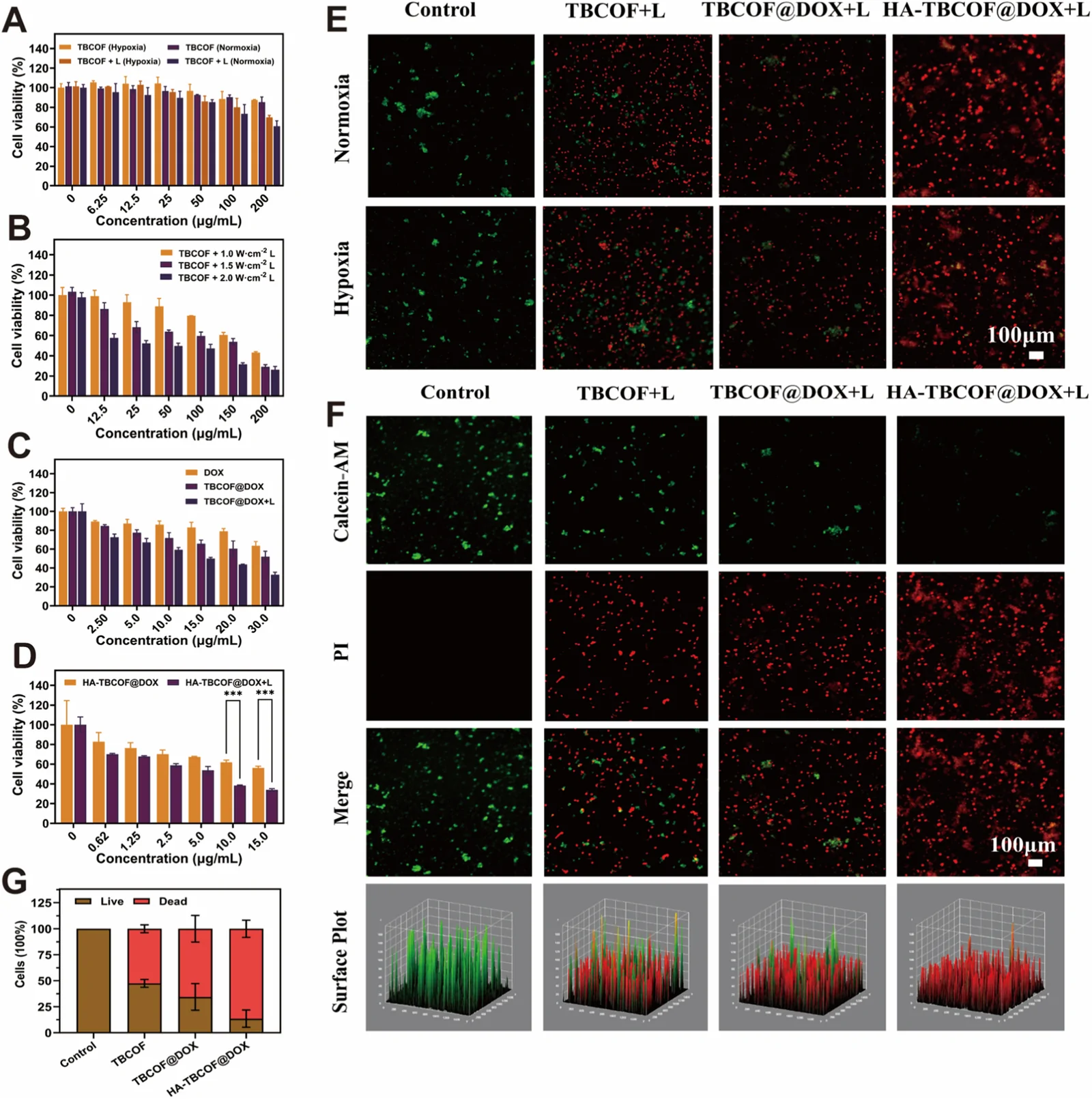
In vitro cytotoxicity and live/dead staining of TBCOF, TBCOF@DOX, and HA-TBCOF@DOX in 4T1 cells with or without 660 nm laser irradiation. (A) Cell viability of 4T1 cells treated with TBCOF at various concentrations under normoxia, hypoxia (1% O_2_), and with laser irradiation (1.0 W cm^−2^). (B) Cell viability of TBCOF with laser irradiation at different power densities (1.0, 1.5, 2.0 W cm^−2^) for photothermal effect optimization. (C) Cell viability of TBCOF@DOX with or without laser (1.0 W cm^−2^) compared to free DOX. (D) Cell viability of HA-TBCOF@DOX with or without laser (1.0 W cm^−2^). (E) PI staining images of 4T1 cells after different treatments (control, TBCOF + L, TBCOF@DOX + L, HA-TBCOF@DOX + L) under normoxia and hypoxia (1% O_2_) (scale bar: 100 μm). (F) Calcein-AM/PI live-dead staining images of 4T1 cells after different treatments (control, TBCOF + L, TBCOF@DOX + L, HA-TBCOF@DOX + L) under normoxia (scale bar: 100 μm). (G) Quantitative analysis of live and dead cell percentages from Calcein-AM/PI staining under normoxia (p-values were calculated by Student's t-test, *p < 0.05, **p < 0.01, ***p < 0.001, n = 5).

Building upon our mechanistic findings regarding cellular uptake, ROS production, and apoptosis, we quantified the antiproliferative activity of these multimodal therapeutic agents via MTT assays. First, we evaluated the cytotoxicity of bare TBCOF under normoxic and hypoxic conditions (Fig. 4A). Without laser irradiation, TBCOF still triggered concentration-dependent cell death via Fenton reactions, confirming its inherent CDT activity. Laser treatment greatly enhanced tumor cell suppression through PDT/CDT synergy, and this effect was further strengthened in hypoxic environments. We then optimized the photothermal (PTT) effect by adjusting laser power densities. As shown in Fig. 4B, higher light power and TBCOF concentration led to stronger cell ablation; cell viability dropped below 30% at 200 μg mL^−1^ under 2.0 W cm^−2^ irradiation, validating its potent PTT performance. We next constructed TBCOF@DOX chemo-phototherapeutic nanoparticles (Fig. 4C). Free DOX induced only mild dose-dependent cytotoxicity. TBCOF@DOX kept cell viability above 60% without light, whereas laser irradiation sharply boosted its anticancer activity. Benefiting from combined PDT, CDT, PTT, and light-triggered DOX release, cell viability decreased to less than 40% at 30 μg mL^−1^. Finally, we synthesized HA-TBCOF@DOX for active tumor targeting (Fig. 4D). This formulation exhibited the most robust cytotoxicity of all groups. Even in the dark, HA-facilitated internalization, spontaneous DOX leakage, and intrinsic CDT caused considerable cell death. Upon laser activation, the combination of HA targeting, PTT ablation, PDT/CDT oxidative injury, and on-demand chemotherapy achieved maximal tumor suppression, with highly significant differences (***p < 0.001).

To further validate these MTT findings, therapeutic performance was assessed via a Calcein-AM/PI live-dead staining protocol under both normoxic and hypoxic conditions. In this assay, viable cells emitted green fluorescence upon Calcein-AM labeling, while necrotic or apoptotic cells showed red signals from PI incorporation. The untreated control exhibited negligible cell death under both normoxia and hypoxia following 660 nm laser treatment, confirming that oxygen deprivation alone does not induce cytotoxicity (Fig. 4E). Notably, the therapeutic efficacy of all treatment groups was largely preserved under hypoxia (1% O_2_) compared to normoxia, with comparable proportions of dead cells observed across corresponding conditions. This insensitivity to oxygen limitation can be attributed to the oxygen-independent Type I ROS generation and intrinsic Cu^2+^-catalyzed Fenton-like reaction for CDT, which remain fully operational irrespective of ambient oxygen tension. Under normoxia, irradiation of TBCOF alone caused a modest elevation in cell mortality, and TBCOF@DOX exposure further amplified this effect in a stepwise manner. Strikingly, HA-TBCOF@DOX under light activation produced the strongest cytotoxic outcome, with the greatest proportion of dead cells observed across the experimental sets (Fig. 4F). Subsequent quantification validated that, upon illumination, HA-TBCOF@DOX triggered markedly elevated apoptosis rates relative to the other variants under both oxygen conditions, supported by significant statistical differences (Fig. 4G). Collectively, these cytotoxicity results from MTT assays and Calcein-AM/PI live-dead staining underscore the remarkable therapeutic potential of HA-TBCOF@DOX, validating the synergistic strategies of targeted delivery, enhanced cellular uptake, PDT, CDT, and light-triggered DOX release for potent tumor suppression, particularly under hypoxic conditions relevant to solid tumors.

### 3.4 In vivo antitumor treatment

**Fig. 5:**
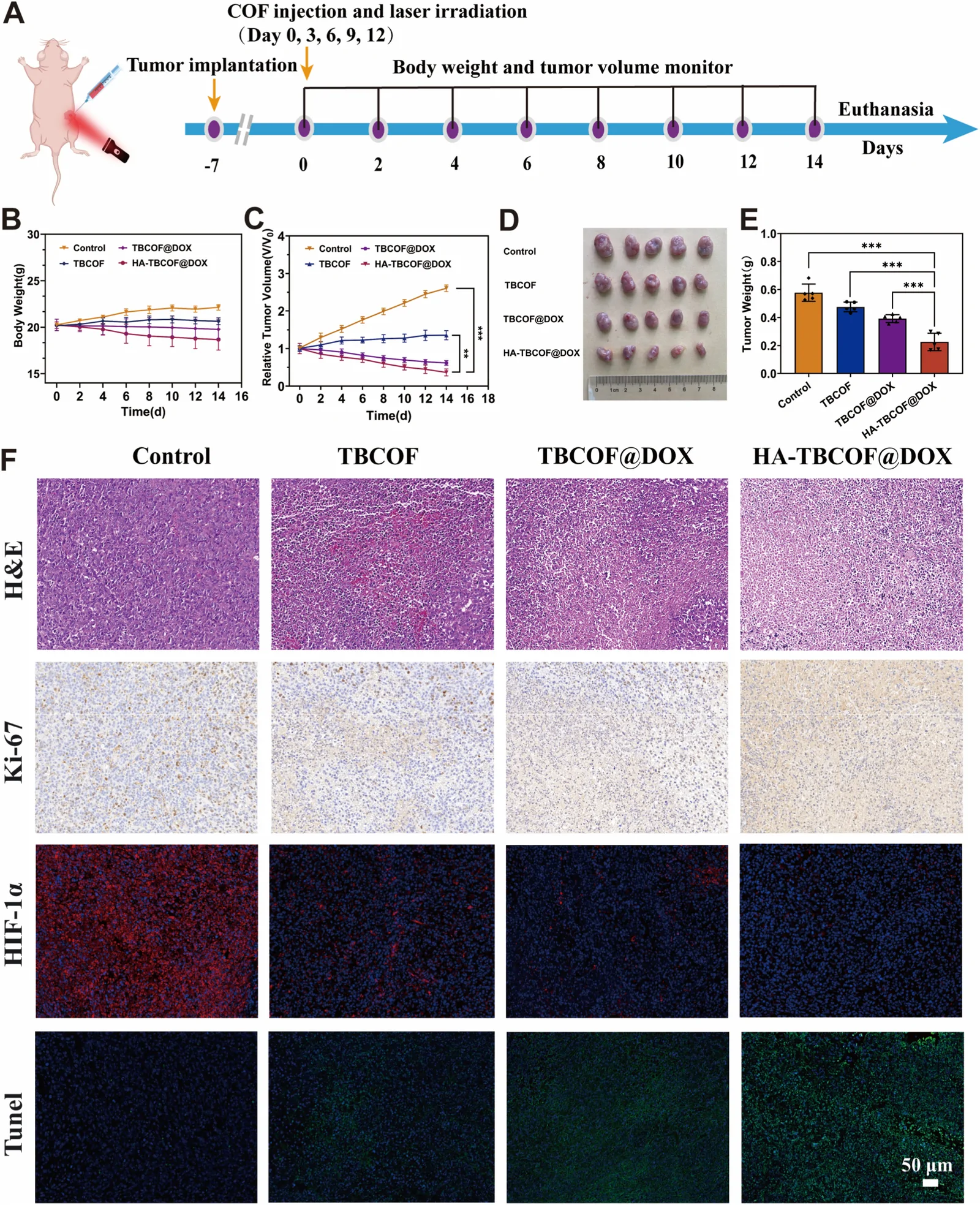
In vivo antitumor efficacy of HA-TBCOF@DOX and control formulations in 4T1 tumor-bearing mice. (A) Schematic protocol of in vivo treatment. (B) Body weight changes of mice during the treatment period (Statistical significance was determined by two-way ANOVA followed by Tukey's multiple comparisons test, n = 5). (C) Tumor volume growth curves during treatment. (D) Ex vivo tumor images after treatment. (E) Tumor weights of each group. (F) H&E staining, Ki67, TUNEL, and HIF-1α immunohistochemistry of tumor sections showing apoptosis, proliferation suppression, and hypoxia alleviation (scale bar: 50 μm).

Given the significant antitumor efficacy of HA-TBCOF@DOX observed in vitro, we established a 4T1 tumor-bearing mouse model to evaluate its in vivo therapeutic performance using the protocol outlined in Fig. 5A. Briefly, mice were randomly assigned to four groups: (1) Control, (2) TBCOF, (3) TBCOF@DOX, and (4) HA-TBCOF@DOX. After injection and subsequent laser irradiation, tumor volumes and body weights were recorded every other day. To assess the biosafety of HA-TBCOF@DOX, body weight changes were monitored throughout the experiment. None of the treatment groups showed obvious weight loss during the 14-day observation period, indicating that the applied doses did not cause significant systemic toxicity under these conditions (Fig. 5B). As shown in Fig. 5C, tumors in the control group grew rapidly, reflecting ineffective tumor control. Both TBCOF and TBCOF@DOX groups exhibited moderate tumor growth inhibition, confirming the antitumor effects of TBCOF and its DOX-loaded counterpart. Notably, the HA-TBCOF@DOX group showed markedly suppressed tumor volumes, even smaller than those in the TBCOF@DOX group, underscoring the benefit of targeted delivery in cancer therapy. Moreover, ex vivo tumor images (Fig. 5D) and weight measurements (Fig. 5E) further confirmed that the HA-TBCOF@DOX group had the smallest tumor size and weight. Collectively, HA-TBCOF@DOX-mediated targeted PDT effectively eliminated tumor cells and restrained tumor progression.

Additionally, H&E staining (Fig. 5F) revealed that both TBCOF@DOX and HA-TBCOF@DOX induced larger necrotic/apoptotic regions in tumors compared to the control and TBCOF groups, with the HA-TBCOF@DOX group exhibiting the most extensive apoptosis. These results further support the synergistic therapeutic approach. Tumor cell proliferation and death were further assessed by Ki67 immunohistochemistry and TUNEL staining. Ki67 is a well-established marker of cell proliferation. The results showed reduced proliferative activity in the TBCOF, TBCOF@DOX, and HA-TBCOF@DOX groups, with the most marked reduction observed in the HA-TBCOF@DOX group, indicating its strongest ability to suppress tumor proliferation under laser exposure. Furthermore, TUNEL staining further revealed enhanced cell death in the TBCOF@DOX and HA-TBCOF@DOX groups, with the latter showing the greatest effect, in line with the H&E findings. Immunostaining for HIF-1α (hypoxia-inducible factor-1α), a key molecular marker of cellular hypoxia, was also performed [49,50]. The results confirmed that TBCOF, TBCOF@DOX, and HA-TBCOF@DOX alleviated intratumoral hypoxia by consuming endogenous H_2_O_2_ and generating O_2_. Taken together, these findings demonstrate that HA-TBCOF@DOX, when exposed to laser, achieves optimal antitumor efficacy through the synergistic strategy of targeted delivery, enhanced cellular uptake, light-triggered DOX release, PDT, and CDT, highlighting its strong potential for cancer therapy.

## 4 Conclusion

In summary, we have successfully constructed a porphyrin-based covalent organic framework via solvothermal polycondensation and further engineered it into HA-TBCOF@DOX nanoplatforms through efficient DOX encapsulation and subsequent hyaluronic acid surface decoration. The resulting nanoplatform possesses a remarkable array of advantages, including exceptionally high drug loading capacity, HA-mediated active tumor targeting, robust photodynamic ROS generation, stable photothermal conversion, pH-responsive DOX release, and intrinsic Cu^2+^-mediated chemodynamic activity proposed to catalyze a Fenton-like reaction to convert endogenous H_2_O_2_ into •OH for CDT, while simultaneously generating O_2_ to relieve tumor hypoxia and enhance PDT, thereby achieving synergistic CDT/PDT. In vitro and in vivo studies confirmed that laser-activated HA-TBCOF@DOX effectively suppressed tumor growth, induced late-stage apoptosis, alleviated tumor hypoxia, and exhibited no apparent systemic toxicity in the 4T1 tumor-bearing mouse model under the examined dose and time frame. Collectively, this work establishes a multifunctional COF-based targeted nanoplatform that seamlessly integrates photodynamic therapy, chemodynamic therapy, photothermal therapy, and chemotherapy, offering a promising strategy that warrants further investigation in additional models for highly efficient cancer treatment.

## CRediT authorship contribution statement

**Yongjie Mo:** Conceptualization, Data curation, Formal analysis, Investigation, Writing – original draft, Writing – review & editing. **Jie Hou:** Supervision, Validation, Visualization. **Hongli Li:** Supervision, Validation, Visualization. **Lijuan Wang:** Data curation, Investigation, Validation, Visualization. **Linlu Zhao:** Data curation, Investigation, Validation, Visualization. **Linhua Zhu:** Formal analysis, Funding acquisition, Resources, Writing – review & editing. **Chunyan Dai:** Project administration, Writing – review & editing.

## Declaration of competing interests

The authors declare that they have no known competing financial interests or personal relationships that could have appeared to influence the work reported in this paper.

## Data Availability

Data will be made available on request.

## References

[1] Bray F., Laversanne M., Sung H.Y.A., Ferlay J., Siegel R.L., Soerjomataram I., Jemal A. (2024). Global cancer statistics 2022: GLOBOCAN estimates of incidence and mortality worldwide for 36 cancers in 185 countries. Ca-Cancer J. Clin..

[2] Siegel R.L., Kratzer T.B., Wagle N.S., Sung H., Jemal A. (2026). Cancer statistics. Ca-Cancer J. Clin..

[3] Cai Y.Y., Chai T., Nguyen W., Liu J.Y., Xiao E.H., Ran X., Ran Y.P., Du D., Chen W., Chen X.Y. (2025). Phototherapy in cancer treatment: strategies and challenges. Signal Transduct. Targeted Ther..

[4] Overchuk M., Weersink R.A., Wilson B.C., Zheng G. (2023). Photodynamic and photothermal therapies: synergy opportunities for nanomedicine. ACS Nano.

[5] Banerjee T., Thawrani K., Pal A.K., Datta A., Dasgupta J., Ghosh S. (2026). Supramolecular assembly of a heavy-atom-free highly efficient self-deliverable photosensitizer for activable photodynamic therapy. Angew. Chem. Int. Ed..

[6] Li X., Lee S., Yoon J. (2018). Supramolecular photosensitizers rejuvenate photodynamic therapy. Chem. Soc. Rev..

[7] Li H., Zheng X., Li J., Liu T., Liu M., Wu J., Liu W., Wang P. (2026). Ultrasound-triggered type I supramolecular photosensitizer for precise in situ chemiluminescence-based photodynamic cyclic therapy. J. Am. Chem. Soc..

[8] Meng X., Han Y., Wang S., Wang X., Zhang Z., Yao S., Wan X., Liu Z., Ge Z., Li L. (2023). Near-infrared photosensitizers adaptive to tumor hypoxic microenvironment for synergistic photothermal-photodynamic and immunotherapy. Nano Today.

[9] Zhuang J., Qi G., Feng Y., Wu M., Zhang H., Wang D., Zhang X., Chong K.C., Li B., Liu S., Tian J., Shan Y., Mao D., Liu B. (2024). Thymoquinone as an electron transfer mediator to convert type II photosensitizers to Type I photosensitizers. Nat. Commun..

[10] Tian J., Li B., Zhang F., Yao Z., Song W., Tang Y., Ping Y., Liu B. (2023). Activatable type I photosensitizer with quenched photosensitization pre and post photodynamic therapy. Angew. Chem. Int. Ed..

[11] Zhu Y., Ling X., Wang X., Liu B. (2026). Excited-State-Guided molecular design system for type I photosensitizers. ACS Nano.

[12] Liu S., Wang Y., Yao S., Li Q., Sun C., Chen Y., Ye J., Li W., Wu D., Zhou J., Cheng H., Ding Y. (2025). Combinative dual-nanoparticle assembly improves oxygen-autarky PDT efficacy in hypoxic tumors. Chem. Eng. J..

[13] Li S., Yao J., Zhang S., Zhou X., Zhao X., Di N., Hao S., Zhi H. (2023). Prognostic value of tumor-microenvironment-associated genes in ovarian cancer. BIO Integration.

[14] Akinyemi A.O., Alam M.R., Howlader M., Farahani M.E., Gu L., Li C., Ngule C., Fulp K., Owoyemi B., Wang X., Nwadialo S., Selani F., Esoe M., Oluwafunminiyi O., Li Z. (2026). Targeting GRP78 and the proteostasis network to break the therapeutic ceiling in neuroendocrine prostate cancer. iNew Medicine.

[15] Li Y., Duan Y., Li Y., Gu Y., Zhou L., Xiao Z., Yu X., Cai Y., Cheng E., Liu Q., Jiang Y., Yang Q., Zhang F., Lei Q., Yang B. (2025). Cascade loop of ferroptosis induction and immunotherapy based on metal-phenolic networks for combined therapy of colorectal cancer. Exploration.

[16] Ma Y.N.S., Sui S., Xu W., Kong L., Wu X., Wu J., Gao Y., Yan T. (2025). Engineered extracellular vesicles: advancing cancer therapy through precision nanomedicine. BIO Integration.

[17] Tan K.T., Ghosh S., Wang Z.Y., Wen F.X., Rodríguez-San-Miguel D., Feng J., Huang N., Wang W., Zamora F., Feng X.L., Thomas A., Jiang D.L. (2023). Covalent organic frameworks. Nat. Rev. Methods Primers.

[18] Singh N., Kim J., Kim J., Lee K.Y.W., Zunbul Z., Lee I.J., Kim E., Chi S.G., Kim J.S. (2023). Covalent organic framework nanomedicines: biocompatibility for advanced nanocarriers and cancer theranostics applications. Bioact. Mater..

[19] Zhou L.L., Guan Q., Dong Y.B. (2024). Covalent organic frameworks: opportunities for rational materials design in cancer therapy. Angew. Chem. Int. Ed..

[20] Huang G.Q., Zhang L.Y., Feng J.H., Wu D., Wu L.B., Pan W.L., Jiang Y., Chen M., Chen J.X., Shui P.X. (2025). Hypoxia-responsive covalent organic framework nanoplatform for breast-cancer-targeted cocktail immunotherapy via triple therapeutic switch mechanisms. Small.

[21] Zhang L., Wang S., Yang Q.C., Song A., Wang Y.Y., Wang W.D., Zhang M.J., Li H., Sun Z.J., Deng H.X. (2025). Radioactive diselenide bonded covalent organic framework. Adv. Mater..

[22] Lin K.P., Wang S.C., Yu S.L., Si W., Yang M.J., Xu N.N., Liu Y., Zheng Y., Zhao S., Shi J.H., Yuan J.T. (2025). Porphyrin-based covalent organic framework with NIR absorption: preparation, hyaluronic acid modification, and cascading a hypoxia-sensitive drug for synergistic therapy of cancer phototherapy/chemotherapy. Int. J. Biol. Macromol..

[23] Zhen W.Y., Kang D.W., Fan Y.J., Wang Z.T., Germanas T., Nash G.T., Shen Q.J., Leech R., Li J.H., Engel G.S., Weichselbaum R.R., Lin W.B. (2024). Simultaneous protonation and metalation of a porphyrin covalent organic framework enhance photodynamic therapy. J. Am. Chem. Soc..

[24] Mo Y.J., Li H.L., Hou J., Zhao H.L., Zhao L.L., Bin Xu B., Zhu L.H., Dai C.Y. (2025). Light-activated fluorinated porphyrin COFs: dual oxygen/drug carriers reshaping tumor hypoxia for precision photodynamic-chemo therapy. Microchem. J..

[25] Zhang Y., Zhang M., Hu X., Hao H., Quan C., Ren T., Gao H., Wang J. (2024). Engineering a porphyrin COFs encapsulated by hyaluronic acid tumor-targeted nanoplatform for sequential chemo-photodynamic multimodal tumor therapy. Int. J. Biol. Macromol..

[26] Feng J., Kong F., Yue W.-S., Yu H., He Z.-L., Zhai Y.-N., Dong Y.-B. (2023). Covalent organic framework-based nanozyme for cascade-amplified synergistic cancer therapy. Sci. China Mater..

[27] Zhang B.G., Su X., Kang J., Chen L. (2026). Covalent organic frameworks as multifunctional nanoplatforms for precise cancer therapy: design principles, applications, and future perspectives. Coord. Chem. Rev..

[28] Sun Q.Q., Song Y.H., Liu R.R., Pan W., Gao Y.N., Li Y.H., Li N., Tang B. (2026). DHA/Fe2+-doped porphyrin COFs enable ROS-amplified multimodal tumor therapy. Chem. Commun..

[29] Wu X., Xu X., Wang H., Huang N. (2026). Donor-Acceptor polyimide covalent organic frameworks for photocatalytic generation of singlet oxygen and superoxide anion radicals. Inorg. Chem..

[30] Zhao C., Shi R., Yang X., Zhao J., Zeng S., Qiu Z., Zhang Y., Liu Q. (2025). Cocatalyst-free hydrogen evolution over benzothiazole-based metal covalent organic framework under visible light. Chem. Eng. J..

[31] Chen W., Yang Z., Xie Z., Li Y., Yu X., Lu F., Chen L. (2019). Benzothiadiazole functionalized D–A type covalent organic frameworks for effective photocatalytic reduction of aqueous chromium(vi). J. Mater. Chem. A.

[32] Pang Y., Lv J., He C.C., Ju C.D., Lin Y.L., Zhang C., Li M. (2024). Covalent organic frameworks-derived carbon nanospheres based nanoplatform for tumor specific synergistic therapy via oxidative stress amplification and calcium overload. J. Colloid Interface Sci..

[33] Chen H., Song J., Wang Y., Wang T., Zhang F., Lv Y., Liu Z., Fu J., Kong X. (2024). “Triple-Hop” nano-bomb combining CDT, PDT and immunetherapy for NIR-triggered cancer therapy. Chem. Eng. J..

[34] Zhu Z.-H., Zhang L., Jia S., Ni Z., Li Y.-L., Zou H.-H., Yang Y., Hu Y., Ding D., Tang B.Z., Feng G. (2025). Nanoscale metal-organic framework leveraging water, oxygen, and hydron peroxide to generate reactive oxygen species for cancer therapy. Adv. Funct. Mater..

[35] Chen J.J., Zhou Q., Cao W.W. (2024). Multifunctional porphyrin-based sonosensitizers for sonodynamic therapy. Adv. Funct. Mater..

[36] Liu X.Y., Zhan W.J., Gao G., Jiang Q.C., Zhang X.P., Zhang H.B., Sun X.B., Han W., Wu F.G., Liang G.L. (2023). Apoptosis-Amplified assembly of porphyrin nanofiber enhances photodynamic therapy of oral tumor. J. Am. Chem. Soc..

[37] Xu P.J., Wen C.C., Gao C.J., Liu H.H., Li Y.S., Guo X.L., Shen X.C., Liang H. (2023). Near-Infrared-II-Activatable self-assembled manganese porphyrin-gold heterostructures for photoacoustic imaging-guided Sonodynamic-Augmented photothermal/photodynamic therapy. ACS Nano.

[38] Liang B., Zhao J., Wang J., Li Y., Han B., Li J., Ding X., Xie Z., Wang H., Zhou S. (2023). Nonlinear optical properties of porphyrin-based covalent organic frameworks determined by steric-orientation of conjugation. J. Mater. Chem. C.

[39] Fu Q., Sun X., Zhang T., Pei J., Li Y., Li Q., Zhang S., Waterhouse G.I.N., Li H., Ai S. (2024). Porphyrin-based covalent organic polymers with customizable photoresponses for photodynamic inactivation of bacteria. Sci. Total Environ..

[40] Chen Y., Jiang D. (2024). Photocatalysis with covalent organic frameworks. Acc. Chem. Res..

[41] Wang J., Qu X., Zhao L., Yan B. (2021). Fabricating nanosheets and ratiometric detection of 5-Fluorouracil by covalent organic framework hybrid material. Anal. Chem..

[42] Li R., Zhu Y.Y., Zhang X.W., Li S.Y., Wang D., Liu Z.P., Wang X.Y., Hou Y.S., Li S.X. (2025). Crystalline porous frameworks (MOF@COF) for adsorption-desorption analysis of β-lactam drugs. Polymer.

[43] Li S.T., Liu Y., Dong G.L., Wang Y.H., Li Y.N., Hu L.F., Yang G.Q. (2026). Rationally designed thiazole-based COF with multiphoton activity for drug delivery and synergistic therapy of deep-seated tumors. J. Nanobiotechnol..

[44] Xu J.Q., Chu T.J., Yu T.T., Li N.S., Wang C.L., Li C., Zhang Y.L., Meng H., Nie G.J. (2022). Design of diselenide-bridged hyaluronic acid nano-antioxidant for efficient ROS scavenging to relieve colitis. ACS Nano.

[45] Kesharwani P., Chadar R., Sheikh A., Rizg W.Y., Safhi A.Y. (2022). CD44-Targeted nanocarrier for cancer therapy. Front. Pharmacol..

[46] Hou X.Y., Zhong D., Chen H.Y., Gu Z.W., Gong Q.Y., Ma X.L., Zhang H., Zhu H.Y., Luo K. (2022). Recent advances in hyaluronic acid-based nanomedicines: preparation and application in cancer therapy. Carbohydr. Polym..

[47] Waterhouse N.J., Sedelies K.A., Sutton V.R., Pinkoski M.J., Thia K.Y., Johnstone R., Bird P.I., Green D.R., Trapani J.A. (2006). Functional dissociation of ΔΨm and cytochrome c release defines the contribution of mitochondria upstream of caspase activation during granzyme B-induced apoptosis. Cell Death Differ..

[48] Yang S., Zhang Z., Dai C., Li J., Tian M. (2023). Visualizing voltage in mitochondria via a unique polarity-responsive fluorescent probe. Chem. Eng. J..

[49] Liu X., Deng H., Tang J., Wang Z., Zhu C., Cai X., Rong F., Chen X., Sun X., Jia S., Ouyang G., Li W., Xiao W. (2022). OTUB1 augments hypoxia signaling via its non-canonical ubiquitination inhibition of HIF-1α during hypoxia adaptation. Cell Death Dis..

[50] Dai W., Guo R., Na X., Jiang S., Liang J., Guo C., Fang Y., Na Z., Li D. (2024). Hypoxia and the endometrium: an indispensable role for HIF-1α as therapeutic strategies. Redox Biol..

