# Supplementary material for: Porphyrin-copper (II) covalent organic framework nanoplatform with intrinsic bifunctional photodynamic and chemodynamic activities for synergistic multimodal cancer therapy
Source: Mater Today Bio. 2026 Aug 17;40:103579. doi: 10.1016/j.mtbio.2026.103579 (PMC13524741; doi:10.1016/j.mtbio.2026.103579)
Supplement: Supplementary file 1 [file mmc1.docx]

Supporting Information

**Porphyrin-Copper (II) Covalent Organic Framework Nanoplatform with Intrinsic Bifunctional Photodynamic and Chemodynamic Activities for Synergistic Multimodal Cancer Therapy**

Yongjie Mo ^a^, Jie Hou ^a, b^, Hongli Li ^a^, Lijuan Wang ^a,c^, Linlu Zhao ^b^, Linhua Zhu ^a,*^, Chunyan Dai ^a,*^

^a^ Key Laboratory of Tropical Medicinal Resource Chemistry of Ministry of Education, Key Laboratory of Tropical Medicinal Plant Chemistry of Hainan Province, The International Joint Research Center for Clean and Efficient Utilization of Hydrocarbon Resources in the South China Sea of Hainan Province, Engineering Research Center of Tropical Marine Functional Polymer Materials of Hainan Province, Key Laboratory of Water Pollution Treatment and Resource Reuse of Hainan Province, Key Laboratory of Functional Organic Polymers of Haikou, Hainan Normal University, Haikou 571158, PR China

^b^ Key Laboratory of Emergency and Trauma of the Ministry of Education, The First Affiliated Hospital, Hainan Medical University, Haikou, 571199, China

^c^ Yazhou Bay Innovation Institute/College of Marine Science and Technology, Hainan Tropical Ocean University, Sanya, Hainan 572022, People’s Republic of China

^*^ Corresponding authors.

E-mail: zhulinhua@hainnu.edu.cn (L. Zhu), [daichunyan@hainnu.edu.cn](mailto:daichunyan@hainnu.edu.cn) (C. Dai)

**1. Supplementary data**

**Fig. S1.** High‑resolution XPS C 1s spectrum of **TBCOF**, showing peaks at 284.80 eV (C–C) and 287.13 eV (C–O).

**Fig. S2.** High‑resolution XPS N 1s spectrum of **TBCOF**, deconvoluted into two components at 397.83 eV (imine C=N) and 400.19 eV (amine C–N).

**Fig. S3.** High‑resolution XPS S 2p spectrum of **TBCOF**, showing S 2p_3/2_ at 164.33 eV and S 2p_1/2_ at 165.62 eV.

**Fig. S4.** High‑resolution XPS Cu 2p spectrum of **TBCOF**, with Cu 2p_3/2_ at 933.69 eV, a satellite peak at 942.00 eV (Cu^2+^), and Cu 2p_1/2_ signals at 953.54 eV and 961.85 eV.

**Fig. S5.** N₂ adsorption‑desorption isotherms of **TBCOF** at 77 K.

**Fig. S6.** NL‑DFT pore size distribution of **TBCOF**, showing a narrow peak centered at 4.220 nm.

**Fig. S7.** Crystal lattice spacing of **TBCOF**.

**Fig. S8.** SEM image of **TBCOF**, showing uniform nanoparticles of approximately 100 nm that agglomerate into a powder‑like morphology.

**Fig. S9.** SEM image of **HA-TBCOF-DOX**.

**Fig. S10.** EDS elemental mapping of **TBCOF**, confirming homogeneous distribution of C, N, O, and Cu.

**Fig. S11.** EDS elemental mapping of **HA‑TBCOF@DOX**, showing enhanced C and N signals from DOX and HA.

**Fig. S12.** DLS size distribution of **TBCOF** in pH 7.4 buffer. Mean hydrodynamic diameter = 283.9 nm.

**Fig. S13.** DLS size distribution of **TBCOF** in pH 5.4 buffer. Mean hydrodynamic diameter = 274.5 nm.

**Fig. S14.** Colloidal stability of **TBCOF** over 6 days in pH 7.4 and pH 5.4 buffers and 10% FBS/PBS, showing nearly unchanged hydrodynamic diameters. Fig. S14.

**Fig. S15.** Hydrodynamic diameters of **TBCOF** in PBS at varying ionic strengths (0.5X, 1X, and 2X), showing marginal size variation and excellent electrolyte tolerance.

**Fig. S16.** DLS size distribution of HA‑TBCOF@DOX. Mean hydrodynamic diameter = 401.5 nm.

**Fig. S17.** Quantitative protein adsorption on **TBCOF** and **HA-TBCOF**, demonstrating ~27% reduction in protein corona formation upon HA modification.

**Fig. S18.** TGA thermograms of **TBCOF** and **HA‑TBCOF@DOX**. Onset decomposition temperatures: 340.17 °C (**TBCOF**) and 329.01 °C (**HA‑TBCOF@DOX**).

**Fig. S19.** EPR spectra of •OH generated by **TBCOF** with the addition of H_2_O_2_ under dark conditions.

**Fig. S20.** SOSG fluorescence intensity of **TAPP‑Cu** (porphyrin monomer) under 660 nm laser irradiation as a function of time (0–10 min).

**Fig. S21.** SOSG fluorescence intensity of **TBCOF** under 660 nm laser irradiation as a function of time (0–10 min).

**Fig. S22.** UV‑Vis absorbance calibration curve of **DOX** at 480 nm (R^2^ > 0.9992).

**Fig. S23.** FT‑IR spectra of **TBCOF@DOX** before and after DOX release at pH 5.4, showing no evident shift or new bands, confirming framework stability.

**Fig. S24.** Digital photo of **HA‑TBCOF@DOX** and **water** after laser irradiation (1.0 W·cm^-2^, 660 nm).

**Fig. S25.** Quantification of thermal intensities of images in a digital photo.

**Fig. S26.** Temperature monitoring of **HA‑TBCOF@DOX** under laser irradiation (125 μg·mL^-1^, 1.0 W·cm^-2^, 660 nm).

**Fig. S27.** Correlation curve of the negative natural logarithm of the cooling period and temperature.

**Fig. S28.** Brightfield images of JC-1-stained 4T1 cells. White-light images acquired after JC-1 staining confirm comparable cell density and morphology across treatment groups, ensuring that observed red/green fluorescence variations reflect genuine ΔΨm changes. Scale bar: 100 μm.

**Fig. S29.** Enlarged CLSM image of DCFH-DA and Hoechst 33342-stained 4T1 cells in the **TBCOF@DOX+L** group.

**Fig. S30.** Enlarged CLSM image of DCFH-DA and Hoechst 33342-stained 4T1 cells in the **HA-TBCOF@DOX+L** group.

**Fig. S31.** Fluorescence intensity line profile of DCFH-DA and Hoechst 33342 across individual cells in the **TBCOF@DOX+L** group.

**Fig. S32.** Fluorescence intensity line profile of DCFH-DA and Hoechst 33342 across individual cells in the **HA-TBCOF@DOX+L** group.
